# Application of Gaussian Mixtures in a Multimodal Kalman Filter to Estimate the State of a Nonlinearly Moving System Using Sparse Inaccurate Measurements in a Cellular Radio Network

**DOI:** 10.3390/s23073603

**Published:** 2023-03-30

**Authors:** Artjom Lind, Shan Wu, Amnir Hadachi

**Affiliations:** Intelligent Transportation Systems Lab, Institute of Computer Science, University of Tartu, Narva Mnt 18, 51009 Tartu, Estonia; artjom.lind@ut.ee (A.L.);

**Keywords:** Kalman filter, Gaussian mixture model, cellular network, geolocation, synthetic data generation

## Abstract

Kalman filter is a well-established accuracy correction method in control, guidance, and navigation. With the popularity of mobile communication and ICT, Kalman Filter has been used in many new applications related to positioning based on spatiotemporal data from the cellular network. Despite the low accuracy compared to Global Positioning System, the method is an excellent supplement to other positioning technologies. It is often used in sensor fusion setups as a complementary source. One of the reasons for the Kalman Filter’s inaccuracy lies in naive radio coverage approximation techniques based on multivariate normal distributions assumed by previous studies. Therefore, in this paper, we evaluated those disadvantages and proposed a Gaussian mixtures model to address the non-arbitrary shape of the radio cells’ coverage area. Having incorporated the Gaussian mixtures model into Switching Kalman Filter, we achieved better accuracy in positioning within the cellular network.

## 1. Introduction

The field of mobile positioning (MP) has evolved since the introduction of location-based services. Initially designed to rely on sparse call detail records (CDR) data, MP offered primitive geo-fencing and later matured into near-real-time positioning and trajectory reconstruction technology with moderate accuracy [1]. The growth of cellular networks, the deployment of new communication standards, and the overall popularity of smartphones contributed to a broad interest in mobility analysis with cellular networks. The general trend toward big-data-related technologies and corresponding data mining studies has caused the field to evolve into a well-established research domain. Numerous use cases illustrate the successful application of cellular network mobility analysis in various areas. Seasonal mobility analysis was one of the first among them due to tolerance to positioning accuracy, availability of many subscribers, and the corresponding long-term historical data [2]. The results showed acceptable accuracy compared to traditional sources (questionnaires, surveys, and population censuses). Based on the obtained results, the method has found further applications in tourism [3], demographic analysis [4], epidemics modeling [5], urban planning [6], and transportation [7].

Mobile positioning accuracy was not critical in earlier studies, yet it became vital in the era of autonomous unmanned vehicles (UAV). Precise localization and tracking are essential aspects of reliable navigation. The MP offered reliable backup in mission-critical scenarios without a global positioning system (GPS) and active/passive sensors malfunction. Otherwise, MP was incorporated in sensor fusion setups offering even better accuracy. Still, MP can hardly compete with the established positioning and tracking technologies, but it can provide a large-scale understanding of mobility and displacement in rural and urban areas [8]. Therefore, it offers excellent potential for further research.

Another area of application of MP is urban planning and smart transport. For instance, the MP has offered a new way of measuring traffic volume in road surveillance and monitoring at a macro-level [9]. An MP-based method produced acceptable results comparable to state-of-the-art methods like inductive loops, cameras, and magnetometer-based devices [10].

From this perspective, this paper explores the potential of increasing mobile positioning accuracy without the augmentation of any other sensors. The approach adopted focuses on introducing a better model for representing the mobile network coverage areas and integrating it with modified switching Kalman filter.

## 2. Related Work and CDR Data Limitations

The moment we are talking about mobile cellular data and mobility. The CDR data is the most explored data for those purposes. The amount and scale of data make it an excellent source for depicting and sensing urban mobility dynamics and intercity travel (ICT). However, this data could be more sparse in time and space. Moreover, the level of accuracy in localization is very scarce, and it contains noise in the trajectories due to the handover process. These challenges and the potential behind this data made it very appealing for researchers to explore its integration into ICT applications and emerging technologies.

The CDR logs are collected on the cellular operator back-end and used for billing purposes; they normally contain privacy-sensitive information and are therefore not exposed for research. The cellular coverage maps reflect the operator’s service area and are not exposed to the public. By combing the CDR data and the cellular coverage map, we can build mobility trajectories reflecting changes in location area in time and space.

Hence, one of the first steps concerning this data type is extracting reliable movement trajectories. For example, in [11], the authors presented a method based on two major blocks that starts by applying a Bayesian-based induction method to perform localization and then reconstruct the trajectories from CDR data using cell coverage overlap filtering ping-pong handover phenomena. The obtained results demonstrated potential improvement compared to the baseline. However, there is still a window for improvement by tuning the Bayesian approach or modeling better the coverage areas.

These extracted trajectories are still not so refined. That’s why most applications focus on the macro-level of analysis and representation. Hence, they are much work on using these data for discovering work and home location areas as presented in [12], where the authors relied on spatiotemporal analysis using clustering techniques and regression to identify home and work location areas. Moreover, CDR data was not only considered for depicting mobility patterns [13] but also as a potential information source to support or replace census data or demographic statistics [14].

Therefore, one of the major contributions that have been made is focused on extracting origin-destination matrices using CDR data as demonstrated in [7,15,16]. For example, in [16], the authors derive their approach to OD-matrix by relying on node-to-node transient OD matrices. An optimization model is used in conjunction with a microscopic simulation to achieve their final results to define the scaling factors that converge to the real observation of traffic counts. The outcome showed the possibility of using CDR data to extract the OD matrices. Still, it has some limitations concerning the scaling method’s simplicity and frequency or density of CDR data at specific locations.

Motivated by the need to localize mobiles in harsh conditions, [17] proposed a Bayesian-based method using CDR data incorporating additional advantageous information such as the distances to the base station, neighbor stations’ information, and signal to interference and noise. The work showed the potential of CDR data for localization. Nevertheless, it still needs to improve compared to GPS positioning.

In addition, to the challenges presented by the sparseness of CDR data, there is a lot of potential and work done behind CDR data. Still, more work is needed in exploring alternative and unorthodox methods to refine the quality of the data and increase its accuracy. Moreover, there is also a need for having a ground truth trajectories dataset for evaluation, such as GPS traces. It is not apparent to always access GPS traces and their corresponding CDR logs due to GDPR regulations which constitutes another challenge to the scientific community working on this topic. Hence, most of the conducted research could only publish the results without permission to publish the used dataset or validation data in some cases.

Nevertheless, some datasets are available in the literature, but they have some limitations. For example, Mobile Data Challenge (MDC) data [18], CRAWDAD ctu-personal (CTU) [19], Orange Data for Development (D4D) Senegal Challenge [20] and MIT’s Reality Mining Dataset (RMD) [21]. Table 1 contains an overview of the available datasets. It is clear that none of those publishes the corresponding radio coverage maps, and only a few publish GPS ground truth. However, coverage map data for corresponding CGI is crucial for estimating the subscriber’s location based on the CDR log. The presence of the GPS ground truth is important to understand the method’s accuracy for getting a clear understanding of the accuracy by computing, for example, the mean square error (MSE) between the location estimated by the method and the actual location recorded by GPS at the time the CDR event was recorded.

Other methods allowing us to estimate the position using Radio Frequency (RF) coverage are based on sequential Monte Carlo family, such as Particle Filters (PF). However, most studies rely on user equipment (UE) related cases: correcting GPS noise, sensor fusion (GPS + IMU), and motion tracking. The CDR-based mobile positioning is in contrast base station (BS) related approach, assuming all the RF coverages are known, and positioning can be performed offline on CDR logs. Studies of this kind are rare, with initial KF attempt [22] illustrating a proof of concept. The PF application of mobile positioning (MP) based on CDR has been considered in [23] for potentially better accuracy to SKF [22]. Yet the resulting accuracy gain was marginal compared to SKF. The performance of PF was significantly lower (considering the large number of particles required for higher accuracy of PF). The third family of methods addressing the subject are conditionally Markov switching hidden linear models (CMSHLM) [24]. Those models allow the calculation of exact solutions of the filter in contrast to the approximate solution of KF or PF. The current paper focused on coverage estimation improvement, and therefore we did not have testing of the RF or CMSHLM in the scope of this paper. We have re-used the SKF and employed Gaussian Mixture Models to perform the RF coverage estimation and provide measurements. The Gaussian Mixture Models are well-known in the field of wireless positioning. Yin et al. employed GMMs to address the non-Gaussian measurement errors in a wireless sensor network (WSN) [25]. Laneuville et. al adopted GMMs to address the exponential growth of the belief state in a multiple model filter [26].

## 3. Methodology

Our Methodology is focused on introducing Gaussian Mixture model (GMM) enhancement of the representation of radio frequency (RF) signal coverage area based on our previous work on Switching Kalman Filter (SKF), which is capable of labeling the CDR records concerning mobility models *Stay* and *Move* correspondingly and positioning the mobile devices within the coverage areas [22].

### 3.1. Kalman Filter

In essence, Kalman’s filter (KF) is a recursive filter performing an estimation of the hidden state of the linear dynamic system (1). The estimated state of a system $x_{t}$ (belief state) at time *t* is represented by continuous random variable $X_{t}$ with multivariate (joint) normal distribution. The corresponding probability is distributed around the mean value $\mu_{t}$ (location) with variation described by $\Sigma_{t}$ (covariance). Here $\mu \in R^{k}$ and $\Sigma \in R^{k\times k}$ where *k* is a number of dimensions in a state vector. Using the inference algorithm KF estimates the belief state at time *t* by applying the transition matrix F to a previously estimated belief state at time t−1, adding transition (process) noise $q_{t}$, which is a zero-mean random variable. The covariance $Q_{t}\in R^{k\times k}$ defines how much noise is added to an estimated belief at time *t*. The estimated belief state is corrected (update) using the evidence $y_{t}$ received at time *t*, where H is an observation model and $r_{t}$ is the measurement noise, a zero-mean random variable (2). The covariance $R_{t}$ defines how precise the measurement is.

(1) $$\begin{array}{c} \begin{array}{c} x_{t}=Fx_{t-1}+q_{t} \end{array}\\ \begin{array}{c} x_{t}∼P\left(X_{t}=x\right)=N\left(x;\mu_{t},\Sigma_{t}\right) \end{array}\\ \begin{array}{c} q_{t}∼N\left(0,Q_{t}\right) \end{array} \end{array}$$

(2) $$\begin{array}{c} \begin{array}{c} y_{t}=Hx_{t}+r_{t} \end{array}\\ \begin{array}{c} r_{t}∼N\left(0,R_{t}\right) \end{array} \end{array}$$

Those routines are the core of KF and may run at independent frequencies $f_{e}$ and $f_{u}$, whereas $f_{e}$ is adjustable and $f_{u}$ is a subject of observation frequency. This simple feature found its application in cases where virtually provide the readings of the sensor in the absence or rarely happening measurements. In this case, the virtual readings (KF estimations) compensate for the lack of actual readings (KF updates). In the subject of navigation, typical use cases of KF include smoothing the fluctuations of GPS readings, filling gaps in rare GPS readings, motion estimation, and collision avoidance [27]. Our initial research on CDR log analysis, however, was focused on subscriber’s essential locations, so-called points of interest (POI), semantic trajectory reconstructions, and subscriber’s mobility prediction [28]. Before conducting the POI discovery, the CDR events in the subscriber’s log have to be filtered concerning the subscriber’s mobility, as those staying in place or moving at pedestrian velocity do contribute to POI discovery. To deduce the subscriber’s possible velocity, we first referred to simple KF modeling subscriber’s velocity as KF’s hidden variable.

(3) $$\begin{array}{c} x_{f}=x_{i}+v_{i}\cdot \Delta t\\ \Delta t=t_{f}-t_{i} \end{array}$$

Initially, we assumed the subscriber’s uniform linear motion used a simple model (3) to describe it. Here $x_{i}$ and $v_{i}$ are the subscriber’s *initial* position and velocity; $x_{f}$ stands for the subscriber’s *final* position in the time interval $\Delta_{t}$, which is the amount of time spent since *initial* till *final* event. From the CDR log, we are only sure about the term *t*, the exact time for an event to occur. The position of the subscriber at time *t* is not known precisely from the CDR event, as it only references the cell where the CDR event was triggered. Yet the CDR events are sparse in time as they are produced only in case of active usage of mobile equipment. Moreover, the radio coverage of the cell may vary from tens of meters to tens of kilometers in radius. The exact shape of the cell’s coverage area is highly amorphous and depends on numerous factors, including terrain, weather, and the number of connected subscribers. The best possible approximation of the coverage is calculated periodically and held on the operator’s side, in raster maps at arbitrary resolution. Those are never exposed to the public due to intellectual property policies. Recently crowd-collected coverage maps are grown in popularity. The subscribers participating in crowd-collecting cellular coverage constantly report their GPS coordinates and connected cell iD. The corresponding coverage area is then formed by grouping the reported GPS locations seen under the same cell iD. The resulting coverage maps are approximated by polygon structures associated with specific cell iD. In our previous studies [22], we relied on crowed collected polygonal data from the cell coverage maps. Initially, we simplified the polygons to circles, assuming the signal is stronger in the center of the polygon and weaker on the periphery.

(4) $$X∼N(\mu ,\sigma^{2})$$

Therefore the location of the subscriber, as well as the cell coverage, are both modeled using normal (Gaussian) random variable (4) in our initial studies, where the location *x* of the subscriber lies within the normal distribution, with mean location *m* and variance $\sigma^{2}$. The variance shows how far the actual location may deviate from the mean location. Considering the assumption a radio signal is stronger in the middle of the cell, the normal random variable *X* is used to describe the coverage area of a specific cell, where μ corresponds to coordinates of the cell’s center, and σ corresponds to the radius of a cell.

(5) $$X_{f}=X_{i}+v_{i}\cdot \Delta t$$

As a result, our uniform linear motion formula (3) does not operate scalars $x_{f}$ and $x_{i}$ but rather normal random variables $X_{i}$ and $X_{f}$ (5); the subscriber’s *initial* and *final* locations.

(6) $$\vec{x}=\begin{array}{c} x\\ y \end{array}$$

(7) $$\vec{v}=\begin{array}{c} v_{x}\\ v_{y} \end{array}$$

Considering the subscriber’s location is given in two-dimensional space, the corresponding *x* and *v* become vectors $\vec{x}$ (6) and $\vec{y}$ (7). Where *x*, *y* are subscriber’s location, coordinates on *x* and *y* axes; $v_{x}$, $v_{y}$ are velocities along *x* and *y* axes.

(8) $$\vec{X}∼N\left(\vec{m_{x}},\Sigma_{x}\right)$$

(9) $$\vec{m_{x}}=\left[\begin{array}{c} m_{x}\\ m_{y} \end{array}\right]$$

(10) $$\Sigma_{x}=\left[\begin{array}{cc} \sigma_{x}^{2} & cov(x,y)\\ cov(x,y) & \sigma_{y}^{2} \end{array}\right]$$

(11) $$cov\left(x,y\right)=p_{x,y}\sigma_{x}\sigma_{y}$$

Taking into account the location given using the vector $\vec{x}$ (5); the normal random variable *X* (4) becomes multivariate $\vec{X}$ with mean location given with 2-dimensional vector $\vec{m_{x}}\in R^{2}$ (9) and *covariance matrix* $\Sigma_{x}\in R^{2\times 2}$ (10); where cov(x,y) is *covariance* and $\sigma_{x}^{2}$, $\sigma_{y}^{2}$ are *variances* of *x* and *y* correspondingly.

(12) $$\vec{X_{f}}=\vec{X_{i}}+\vec{v_{i}}\cdot \Delta t$$

**Figure 1:**
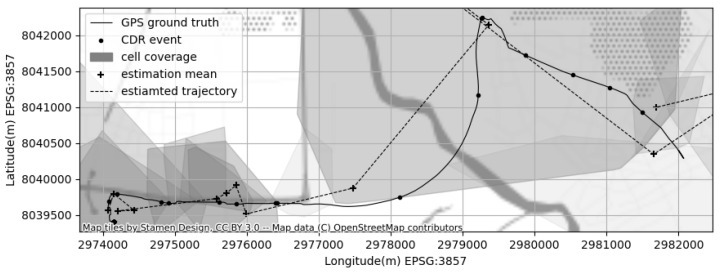
An example of CDR based localization; the method estimates the location based on cell coverage areas using Kalman-Filter.

The uniform linear motion model becomes Equation (12). Figure 1 illustrates the sample CDR log, the approximate cell coverage areas, and the resulting KF approximation of the location for each CDR event.

Originally Kalman’s filter was designed for real-time scenarios where historical data is unavailable, and KF must gain estimation confidence based on evidence arriving in real time. Access to the live stream of CDR logs would assume integration with the corresponding cellular network stack and therefore seemed unfeasible at the time of the research. The CDR logs serve billing purposes and are essentially based on calculating the amount of mobile usage. Therefore those are collected periodically into archive files and are available post-factum. The availability of historical data in the form of CDR log archive files allows to feature of the KF with smoothing to compensate the KF gain lags and calculate the optimal estimates $x_{t|T}$ for the whole period of *T* (all available CDRs in historical data of a subscriber).

### 3.2. Smoothing

Rauch-Tung-Striebel (RTS) smoother introduces the backward pass in addition to regular KF forward pass (estimate and update steps), assuming the results of KF forward pass collected and available for a backward pass [29]. In the backward pass, each future estimation $p(x_{k+1}|x_{k})$, future updated estimation $p(x_{k+1}|y_{1:k})$, future smoothed estimation $p(x_{k+1}|y_{1:T})$ and current updated estimation $p(x_{k}|y_{1:k})$ are used to calculate the current smoothed value of the estimation $p(x_{k}|y_{1:T})$; here *T* denotes the estimation is done relying upon all previous data, hence the *k* running backwards k=T..1. Moreover, calculating each *k*-th smoothed value assumes processing all previously collected data (integration part).

(13) $$\begin{array}{c} \begin{array}{c} p\left(x_{k}|y_{1:T}\right)=p\left(x_{k}|y_{1:k}\right)\int \left[\frac{p\left(x_{k+1}|x_{k}\right)p\left(x_{k+1}|y_{1:T}\right)}{p(x_{k+1}|y_{1:k})}\right]dx_{k+1} \end{array} \end{array}$$

Applying the KF on simple CDR it became clear that the sparsity of the CDR data does not allow KF to converge with lower gain $K_{t}$; the KF updates occurred rarely and were not following any linear pattern. The resulting velocity curve was far from smooth, with very low confidence (table). Apparently, the density of the CDR log was not enough for KF to gain confidence around specific velocity values; instead, each new KF update disregarded previously gained confidence. We concluded it was unreasonable to continue with simple KF and uniform linear motion. However, the results suggested we could focus on specific velocity ranges, restricting the KF in adjusting to updates. We experimented with KF and various *F* settings on the same CDR log; once we figured out values that produce higher confidence for specific intervals in CDR log. We call those intervals the mobility episodes, and corresponding *F* matrix values describe the ability to move (aka. mobility models). Finally, we came to the idea of implementing switching between the multiple models within KF filter, following Murphy’s proposal [30].

### 3.3. Switching Kalman Filter with GMM Cell Coverage Enhancement

**Figure 2:**
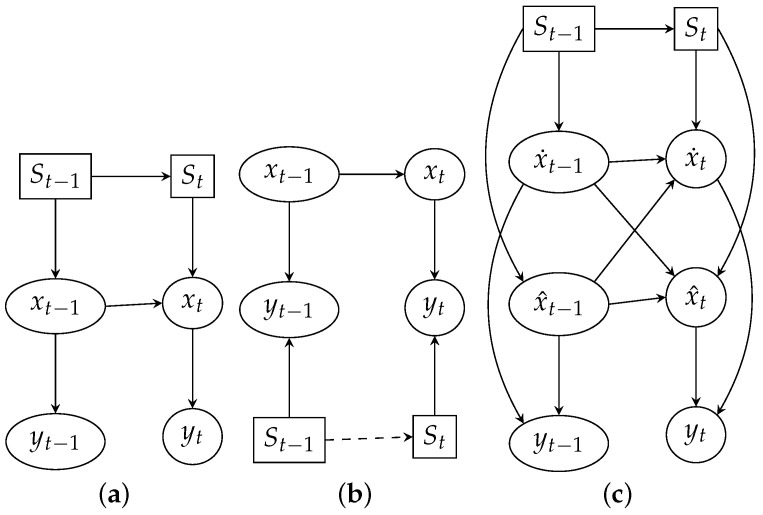
Mixture of Kalman filters; discrete variables are drawn in squares, Gaussian variables are drawn in ellipses. (**a**) Switching estimations; (**b**) Switching observations; (**c**) Switching estimations with factored state.

Switching Kalman Filter (SKF) assumes multiple linear models *M* perform the state estimation in an interleaved manner, a discrete switch variable $S_{t}$ defines which $F_{M}$ and $Q_{M}$ matrices to use to estimate the state $x_{t}$ according to chosen model $M_{t}$ (Figure 2a). In the case of multiple evidence models *M*, the switch variable $S_{t}$ defines which $H_{M}$ and $R_{M}$ matrices to use (Figure 2b) to model the observation. In case we are uncertain about which model *M* to be used at each time step *t* to estimate the state $x_{t}$, the mixture of *M* Gaussians is used to model the state $x_{t}$. The transition matrix *Z* is responsible for Markovian dynamics of $S_{t}$ and defines the model transition probability:

(14) $$\begin{array}{c} Z\left(i,j\right)=P\left(S_{t}=j|S_{t-1}=i\right) \end{array}$$

Having a number of models $N_{m}$, we define the probability of switching from a model *i* at time t−1 to a model *j* at time *t* as follows:

(15) $$\begin{array}{c} Z\left(i,j\right)=\left\{\begin{array}{cc} 0.8 & \mathrm{if}\;i=j\\ \frac{0.2}{N_{m}-1} & \mathrm{otherwise} \end{array}\right. \end{array}$$

giving a higher chance to stay in the same model. The probability of each model $M_{i}$ at time *t* is then calculated given the observation actual at *t*

(16) $$\begin{array}{c} M_{t|t}\left(i\right)=P\left(S_{t}=i|y_{1:t}\right) \end{array}$$

and the probability distribution of the corresponding hidden state variable

(17) $$\begin{array}{c} P\left(x_{t}|S_{t}=i,y_{1:\tau }\right)=N\left(\mu_{t|\tau }^{i},\Sigma_{t|\tau }^{i}\right)\;\;\;\tau \in t,T \end{array}$$

**Figure 3:**
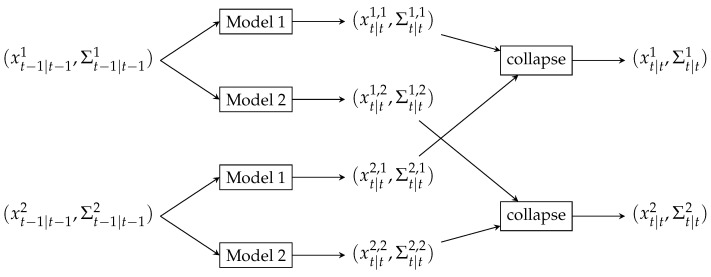
Second order Generalized Pseudo Bayesian collapse strategy using two models. Having the initial belief state estimated by a mixture of two Gaussians at t−1, the posterior belief at *t* is represented by a mixture of four Gaussians (taking into account model switching probability). Finally, four Gaussians are collapsed back to two following moment matching.

In case our initial belief at t−1 is based on two linear models, the corresponding state is given by a mixture of two Gaussians ${\dot{x}}_{t-1}$ and ${\hat{x}}_{t-1}$. Each of them is propagated through two different state estimation equations (one for each model), resulting in the belief state grown by factor 2 at step *t* (Figure 2c). Hence an exponential growth of the belief state with time which is addressed using *collapsing* strategy with Generalized Pseudo Bayesian of order 2 (GPB2) algorithm [31] resulting in $M^{2}$ filtering equations needed at each transition (Figure 3). The Interacting Multiple Models (IMM) would give even better performance with only *M* filtering equations per transition. However smoothing, in this case, would be impossible. Finally, we can define the combined belief of a hidden state $x_{t}$ by the composition of the state estimations given by all models weighed by probabilities of those models

(18) $$\begin{array}{c} P\left(X_{t}|y_{t:\tau }\right)=\sum_{i}M_{t|\tau }\left(i\right)\cdot P\left(X_{t}|S_{t}=i,y_{1:\tau }\right) \end{array}$$

Which is, in essence, a mixture of Gaussian representations of a hidden state, estimated by a hidden combination of multiple linear models. Finally the SKF as a stochastic system with multiple models governing the interactions among the different stochastic quantities: 

(19) $$\begin{array}{c} p\left(s_{t+1},x_{t+1},y_{t+1}|s_{t},x_{t},y_{t}\right)=p\left(s_{t+1}|s_{t}\right)p\left(x_{t+1}|s_{t},x_{t}\right)p\left(y_{t}|x_{t}\right) \end{array}$$

Taking into account the the subscriber state at time *t* is specified using location vector ${\vec{x}}_{t}$ (6) and velocity vector ${\vec{v}}_{t}$ (7), and the mobility follows the equations:

(20) $$\begin{array}{c} {\vec{x}}_{t}={\vec{x}}_{t-1}+{\vec{x}}_{t-1}\Delta t+{\vec{q}}_{t}^{x}\\ {\vec{v}}_{t}={\vec{v}}_{t-1}+{\vec{q}}_{t}^{v} \end{array}$$

we define the subscriber’s hidden state random variable at time *t* as follows:

(21) $$\begin{array}{c} x_{t}∼N\left(\begin{array}{c} {\vec{x}}_{t}\\ {\vec{v}}_{t} \end{array},\Sigma_{t}\right)=N\left(\begin{array}{c} {\dot{x}}_{t}\\ {\hat{x}}_{t}\\ {\dot{v}}_{t}\\ {\hat{v}}_{t} \end{array},\begin{array}{c} \begin{array}{c} \Sigma_{t} \end{array} \end{array}\right) \end{array}$$

where ${\dot{x}}_{t}$, ${\hat{x}}_{t}$ are defining mean subscriber location at time *t* (*longitude*, *latitude*) in meters following EPSG:3857 (Pseudo-Merkator projected coordinate system: https://epsg.io/3857 (accessed on 11 January 2023)) and ${\dot{v}}_{t}$, ${\hat{v}}_{t}$ are defining mean velocity along *longitude* and *latitude* (meters per second). The $\Sigma_{t}$ is a 4×4 matrix and reflects the corresponding covariance between each pair of elements in a random vector. The mobility models used in SKF we define as k∈{Stay,Move} having the initial model transition $Z_{0}$ matrix and initial probabilities $P(S_{0}=k|x_{0})$ of each model *k* where $x_{0}$ is initial state:

(22) $$\begin{array}{cc} \begin{array}{c} Z_{0}=P\left(S_{t}=j|S_{t-1}=i\right)=\left(\begin{array}{cc} 0.8 & 0.2\\ 0.2 & 0.8 \end{array}\right) \end{array}\; & \begin{array}{c} P\left(S_{0}=i\right)=\left(0.5,0.5\right) \end{array} \end{array}$$

The transition matrices *F* for corresponding mobility models are defined as follows:

(23) $$\begin{array}{cc} F^{Stay}=I\; & F^{Move}=\left(\begin{array}{cccc} 1 & 0 & \Delta t & 0\\ 0 & 1 & 0 & \Delta t\\ 0 & 0 & 1 & 0\\ 0 & 0 & 0 & 1 \end{array}\right) \end{array}$$

The corresponding transition noise matrices *Q* are diagonal as we assume no linear relationship between *longitude* and *latitude* in the location ${\vec{x}}_{t}$ as well as in the velocity ${\vec{v}}_{t}$ vectors

(24) $$\begin{array}{cc} Q^{Stay}= & Q^{Move}=\left(\begin{array}{cccc} \sigma_{lon}^{2} & 0 & 0 & 0\\ 0 & \sigma_{lat}^{2} & 0 & 0\\ 0 & 0 & \sigma_{vlon}^{2} & 0\\ 0 & 0 & 0 & \sigma_{vlat}^{2} \end{array}\right) \end{array}$$

where location noise is defined by standard deviation $\sigma_{lon}$ vertical and $\sigma_{lat}$ horizontal (meters, following EPSG:3857). Considering the mobility models *Stay* and *Move* we set the location transition noise low to allow *Stay* model to gain on intervals where the subscriber is switching subsequently between the cell of the same site (multiple cells covering the same area). In this case, the $F^{Stay}=I$ state transition matrix will disregard the velocity and will only consider a drift in a location in any direction with maximal distance reachable with the velocity of walking. In contrast, the velocity noise $\sigma_{vlon}$ vertical and $\sigma_{vlat}$ horizontal (meters per second) is defined with a higher value allowing *Move* model to alter the velocity in broader scope but following the direction of the movement. The $F^{Move}$ state transition matrix takes into account the subscriber’s velocity vector and alters the location according to Equation (20). In addition, the transition noise $Q^{Move}$ adds the drift in location (same way *Stay* model does) and allows the velocity to be altered in broader scope by growing the corresponding values in a covariance matrix, Equation (1). The location noise is calculated as follows:

(25) $$\begin{array}{c} \sigma_{lon}=\sigma_{lat}=V^{Stay}\Delta t \end{array}$$

where $V^{Stay}$ is a walking velocity of 5 km/h (1.39 m/s) and Δt is the time spent since the last KF estimation (seconds). The velocity noise is set to constant, considering the vehicles can accelerate/decelerate rapidly:

(26) $$\begin{array}{c} \sigma_{vlon}=\sigma_{vlat}=20\;\mathrm{km}/h\;\left(5.6\;m/s\right) \end{array}$$

The SKF requires the hidden state variable to be initialized at time t=0 in order to start the inference, and since we have no prior knowledge of the subscriber’s location we use the coverage area of the first seen cell according to the subscriber’s CDR log. We perform Maximum-Likelihood-Estimate (MLE) on cell RF signal pattern from the corresponding raster map to determine the parameters $\vec{\mu }$ and Σ of a probability distribution:

(27) $$\begin{array}{cc} \vec{\mu }=\frac{1}{N}\sum_{i=1}^{N}\vec{x_{i}} & \Sigma =\frac{1}{N}\sum_{i=1}^{N}\left({\vec{x}}_{i}-\vec{\mu }\right){\left({\vec{x}}_{i}-\vec{\mu }\right)}^{T} \end{array}$$

where *N* is an amount of RF signal value pixels in a raster map, $\vec{x_{i}}$ is RF pixel’s coordinate (*longitude*, *latitude*) in meters (following EPSG:3857). The resulting $\vec{\mu }$ and Σ are the parameters of the multivariate normal distribution. The initial belief state of the subscriber’s location $x_{0}$ is then assigned identically for both models:

(28) $$\begin{array}{c} x_{0}^{Stay}=x_{0}^{Move}∼N\left(\begin{array}{c} \mu_{lon}\\ \mu_{lat}\\ 0\\ 0 \end{array},\begin{array}{cccc} \;\Sigma & & 0 & 0\\ & & 0 & 0\\ 0 & 0 & \sigma_{vlon}^{2} & 0\\ 0 & 0 & 0 & \sigma_{vlat}^{2} \end{array}\right) \end{array}$$

where $\mu_{lon}$ and $\mu_{lat}$ is the mean vector $\vec{\mu }$ we obtained in Equation (27) and Σ is the corresponding covariance matrix. As a result, the initial belief state of the subscriber’s location is set to the RF signal distribution of the first cell in the CDR log. We have no information on the initial subscriber’s velocity, therefore we initialize it to 0 in the initial belief state. However, we allow it to be altered in the scope of $\sigma_{vlon}$ vertical and $\sigma_{vlat}$ horizontal velocity standard deviation used in transition noise matrices *Q*, Equation (24).

The observation model is not a subject of switching, and therefore same evidence transform matrix *H* and observation noise *R* is used for all models in SKF. The observation $y_{t}$ and observation noise $R_{t}$ at time *t* is modeled by estimating the RF signal distribution of the visited cell at time *t*. In particular, we consider the RF signal raster map contributing the *N* samples of the RF signal strength ${\vec{x}}_{i}$, where i∈{1…N}. Taking the samples from the raster map, we perform Expectation-Maximization (EM) to estimate the joint distribution of the corresponding mixture of Gaussians. First, we define the levels of the signal strength into *D* groups, having i∈{1…D}, where each $d_{i}$ represents the RF signal strength level. The probability density p(x) of the RF signal strength distribution is defined as follows:

(29) $$\begin{array}{c} p\left(x\right)=\sum_{i=1}^{D}\pi_{i}N\left(x;{\vec{\mu }}_{i},\Sigma_{i}\right) \end{array}$$

where $N(x;{\vec{\mu }}_{i},\Sigma_{i})$ is multivariate normal density of the *i*-th component, ${\vec{\mu }}_{i}$ is a mean vector, $\Sigma_{i}$ is a covariance matrix and $\pi_{i}$ is a mixing probability of the *i*-th Gaussian. The observation $y_{t}$ at time *t* becomes then a mixture of *D* Gaussians, hence represented by *D* mean locations ${\vec{\mu }}_{t}^{i}$ and $\Sigma_{t}^{i}$ covariance matrices weighed by $\pi_{t}^{i}$, where i∈{1..D}. In the KF error calculation step we calculate *D* mean errors $e_{t}^{i}$, error co-variances $E_{t}^{i}$ and Kalman gains $K_{t}^{i}$ using a previous state estimate $x_{t|t-1}$:

(30) $$\begin{array}{c} e_{t}^{i}={\vec{\mu }}_{t}^{i}-Hx_{t|t-1}\\ E_{t}^{i}=H\Sigma_{t|t-1}H^{T}+W_{t}^{i}\\ K_{t}^{i}=\Sigma_{t|t-1}H^{T}{\left(E_{t}^{i}\right)}^{-1} \end{array}$$

where $W_{t}^{i}$ serves as *R* observation noise an observation translation matrix *H* is simply discarding the velocity components of the mean location vector $x_{t|t-1}$:

(31) $$\begin{array}{c} H=\left(\begin{array}{cccc} 1 & 0 & 0 & 0\\ 0 & 1 & 0 & 0 \end{array}\right) \end{array}$$

In the KF update step (2) we calculate *D* the corrected location means $x_{t|t}^{i}$ and co-variances $\Sigma_{t|t}^{i}$:

(32) $$\begin{array}{c} \begin{array}{c} x_{t|t}^{i}=x_{t|t-1}+K_{t}^{i}e_{t}^{i} \end{array}\\ \begin{array}{c} \Sigma_{t|t}^{i}=V_{t|t-1}-K_{t}^{i}E_{t}^{i}{\left(K_{t}^{i}\right)}^{T} \end{array} \end{array}$$

resulting in an updated belief state $x_{t}$ represented by a mixture of *D* Gaussians:

(33) $$\begin{array}{c} \begin{array}{c} p\left(x_{t|t}\right)=\sum_{i=1}^{D}\pi_{i}N\left(x_{t|t};x_{t|t}^{i},\Sigma_{t|t}^{i}\right) \end{array} \end{array}$$

**Figure 4:**
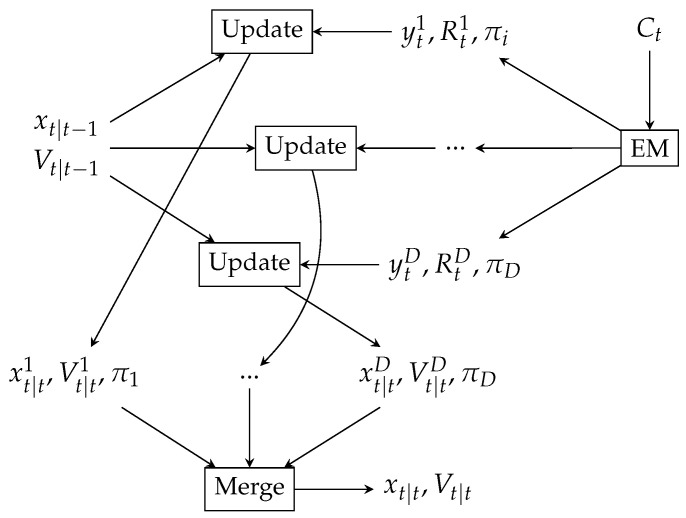
Update the state estimate by applying evidence modeled by Gaussian Mixture; EM estimating RF signal distribution from cell $C_{t}$ visited at time *t*; updating the state estimation $x_{t|t-1}$ using Gaussians from the mixture; merging resulting Gaussian Mixture.

Finally we reduce Gaussian mixture following the Progressive Gaussian Mixture Reduction method (PGMR) [32] and merge to a multivariate normal variable (Figure 4).

(34) $$\begin{array}{c} \pi_{merged}=\Sigma_{i=1}^{C}\pi_{i}\\ \mu_{merged}=\frac{1}{\pi_{merged}}\Sigma_{i=1}^{C}\pi_{i}\mu_{i}\\ V_{merged}=\Sigma_{i=1}^{C}\frac{\pi_{i}}{\pi_{merged}}\left(V_{i}+\left(\mu_{i}-\mu_{merged}\right){\left(\mu_{i}-\mu_{merged}\right)}^{T}\right) \end{array}$$

In particular, PGMR performs in several stages; first, the Runnal’s reduction [33] is performed to calculate Kullback-Leiber (KL) divergence [34] between the original mixture and the target reduced mixture; subsequently the *k*-means clustering of the merged states is performed having KL-divergence as the distance measure. Finally, estimates are refined using iterative optimization using Integral Squared Error (ISE) cost function [35].

## 4. Evaluation and Results

In the following section, we provide the achieved results and compare them with our baseline methods [36]. The method is designed to process the CDR logs assuming that a corresponding cellular coverage map is available and that the cell ids from CDR events are present in the coverage map. In addition, we need ground truth, such as associated GPS traces to the recorded CDR data, to evaluate our approach. As we mentioned in the literature, there is a limitation in finding good datasets for testing and evaluation, and each one has some downsides, as illustrated in Table 1. Therefore, we created a collector mobile application that records the CGI and corresponding GPS at specific time intervals and on the subscriber’s phone activity (call-in, call-out, message-in, message-out, packet data). The collected data is then uploaded to a server back-end for further analysis. On the server side, the collected CGIs events are matched against the cellular coverage map data. As a result, we collected a dataset like the CDR log’s structure, with the extra feature of collecting at specific time intervals unrelated to subscriber activity (the authentic CDR log is moderated by subscriber’s activity only). The corresponding dataset is referred to as CDR’17.

### 4.1. Synthetic CDR Dataset Based on CDR’17

The goal of our research was to prove the advantage of using Gaussian Mixture Models in contrast to simple Gaussian distribution in modeling the signal coverage area. In particular, we integrated both modeling techniques into CDR based mobile positioning method [22] and we run both versions of the method to estimate the locations from the same CDR dataset. Finally we compared the estimated location to actual locations obtained by GPS at the time CDR event was triggered. Before we proceed to discuss the results, we explain how the evaluation dataset was prepared. Since focused on coverage modeling techniques as a possible contributor into positioning accuracy, we skipped collecting the massive real-life CDR and used so called synthetic CDR log we generated. Another argument towards synthetic CDR was unrealistic effort on provisioning large CDR archives with temporally precise GPS ground truth.

Finally, among well known CDR datasets there is none that offers the details we find important: CDR-GPS time synchronized ground truth and real world CGI in CDR headers (see Table 1). Our own collected dataset CDR’17 has only a few unique subscribers. The CDR based mobile positioning method is using multi-modal Kalman Flter (SKF) which allows to focus on various mobility patterns such as: moving directed or chaotically; moving fast or slow at constant velocity; accelerating or decelerating and finally standing in place or almost not moving.

The evaluation dataset shall include examples of all those mobility patterns. In order to achieve it we referred to micro-simulation of a traffic flow composing it by numerous individual trajectories and having the amount of participants correspond to desired amount of mobile subscribers in the required CDR dataset. We deployed open-source package Simulation of Urban Mobility (SUMO) [37] as it allowed customization of mobility profiles as well as offered predefined set of modes (pedestrian, car, bicycle, etc.); in addition it supported modeling the transportation demand based on flow definitions and origin destination matrices, which allowed us make the traffic time distribution more realistic and less uniform. The SUMO allows to record the state of an atomic participant over arbitrary time period (down to 1 s in resolution); effectively solving a task of massive GPS collection as it allows to produce hours of simulation-time GPS data in matter of minutes. In addition to exact location we set SUMO to collect the participant’s velocity, acceleration and direction; allowing us to fully evaluate the KF produced state estimates.

**Figure 5:**
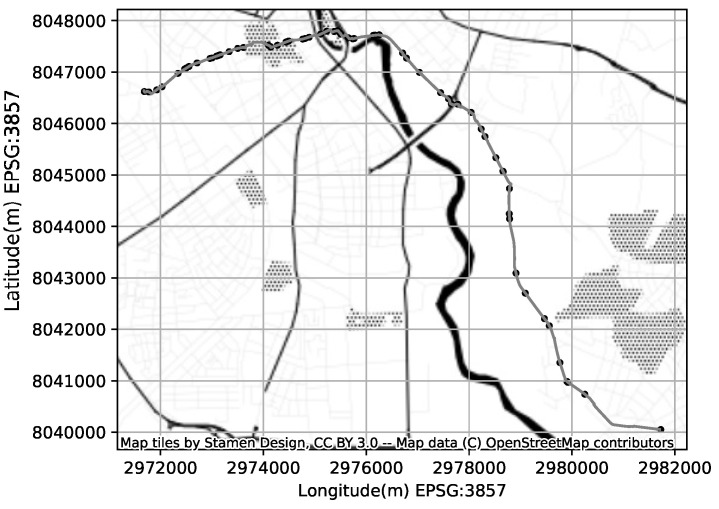
And example of a generated GPS ground truth trajectory (bold gray line) and the corresponding CDR events occurrences (black circles).

The CDRs are triggered in response to subscribers activity and do not reflect the network’s internal events. The visitor location registry records (VLR) however reflect the network internal events related to subscriber’s mobility. In case subscriber is on-call and mobile the VLR will reflect an event each time subscriber connects to a new transceiver while moving. In addition the VLR would reflect a periodic update event to ensure subscriber is still within the are of the last visited transceiver. Since our objective is to evaluate the new cell coverage modeling technique within the SKF, we did not try to mimic the cellular network stack behaviour or adopt the open-source 2g/3g stack like osmocom (Open source mobile communications. Provides software and tools implementing a variety of mobile communication standards: https://osmocom.org (accessed on 11 January 2023)). Instead, we implemented the CDR generator that uses our previously prepared synthetic GPS logs (SUMO). For each GPS point we estimated the strongest reachable cell from the coverage map using euclidean distance to cell’s centroid. As a result we get a sequence of CGIs associated with each GPS point. Finally we reduce the CGI sequences filtering the subsequent equal CGIs, and provision the timestamp and subscriber id from GPS log; leading to a sparse CDR log with the GPS ground truth (Figure 5).

### 4.2. Evaluation

The resulting synthetic CDR log is our validation dataset we used as an input to proposed mobile positioning method (and it’s variants). In our previous research we have shown the accuracy of the SKF method depends on the area of the cell as well as from the variety of visited cell in the individual CDR log [22,38]. Therefore, we designed the CDR generator to maintain the frequency of CDR similar to real world CDR, but at the same time we did not model the subscriber’s phone usage behaviour. The amount of CDR produced by a generator for single subscriber depends on amount of overlaying cell coverage areas the mobile subscriber traverses, triggering a CDR event each time a stronger signal is available.

In contrast to synthetic GPS and CDR logs, the cell coverage map and the corresponding geographical area of interest are based on real world locations. In particular we used the vector map of the city of Tartu (Estonia) as base for the GPS log simulation we performed in SUMO. The corresponding vector map we obtained from up-to date OpenStreetmap (OpenStreetMap is built by a community of mappers that contribute and maintain data about roads, trails, cafés, railway stations, and much more, all over the world: https://www.openstreetmap.org (accessed on 11 January 2023)) (OSM) crowd-collected repository. Next we obtained the cellular coverage map for a specific region defined by the boundary of the previously mentioned vector map of Tartu. We did not cooperate with any of the cellular operators available in Estonia to acquire the cellular coverage map. Instead we referred to crowd-collected cellular coverage repository CellMapper (Signal Tiles and Towers CellMapper: https://www.cellmapper.net (accessed on 11 January 2023)) and extracted the coverage map related to our area of interest. CellMapper is a crowd-collected resource allowing subscribers to contribute their GPS location along with mobile cell id and corresponding signal strength, frequency, generation standard and other related meta information. Having enough of cell signal measurement collected the CellMapper offers an approximate coverage area polygon for a specific cell in addition to cell metadata.

**Figure 6:**
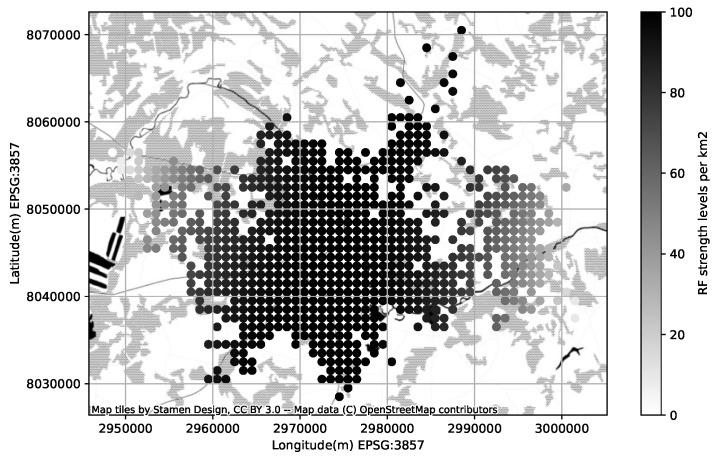
RF signal strength distribution per square kilometer.

**Figure 7:**
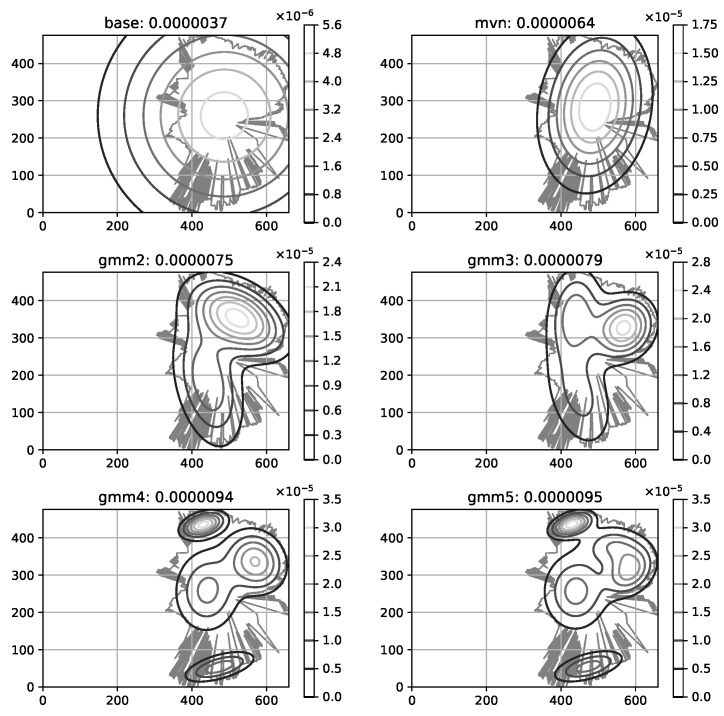
Cell coverage modeling accuracy; an original RF signal coverage area is reflected by a fuzzy polygon; an estimated RF signal distribution is illustrated using contour lines; *base* is the previously used coverage modeling method, *mvn*—a single Gaussian multivariate, *gmmN* Gaussian Mixture Models, where *N* is the number of components.

The mobile operators offering service in the area of interest have more-less equivalent share of RF samples available in CellMapper; for our experiment we have chosen one randomly. One can estimate the confidence of the suggested coverage area offered by a service looking at the amount measurement samples collected for a specific cell; more samples lead to more realistic coverage map (Figure 6). The coverage area polygons provided by CellMapper do indicate an area with an acceptable signal strength, however GPS samples used to estimate a polygon do contain the signal strength information. Having the signal strength samples along with a coverage area polygon makes is possible to initialize the GMM to describe the signal strength distribution (Figure 7).

**Figure 8:**
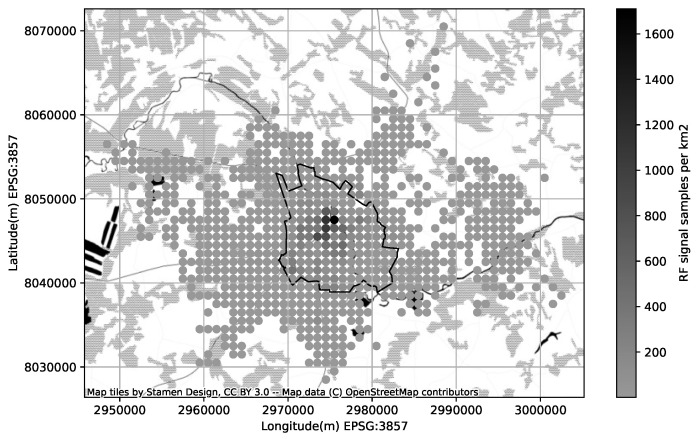
Confidence of the crowd-collected RF signal samples (cellmapper.net (accessed on 11 January 2023)), the circles on the map represent the amount of RF signal samples collected per square kilometer, and the polygon represents the boundaries of Tartu city.

In case a cell has a low confidence due not not enough samples, the coverage area can be calculated from the cell metadata and underlying terrain map (explained in previous section). For our experiment with a synthetic CDR we kept only high-confidence cells, ensuring there there is no zero-coverage areas left in our area of interest (Figure 8). As an additional reliability metrics for each point from the GPS log we calculated the likelihood of being within the area of an acceptable signal strength. In the experiment part we run SKF method and estimate the subscriber location based on provided synthetic CDR dataset and following the coverage map we prepared.

Two versions of coverage modeling techniques were tested: original one with each cell modeled by a multivariate random variable *skf_base* and an improved one using Gaussian Mixture Models *skf_gmm*. The SKF are the estimations of the subscriber location at specific time (CDR event), an estimations are multivariate normal variables, hence including the mean location (*longitude* and *latitude* ) as well as uncertainty given by covariance matrix. In addition the amount of estimations for a single CDR event is equal to amount of mobility models the SKF was initialized to.

The results include likelihood of each estimate to follow the model used by SKF. Evaluating the CDR based MP we calculated root mean square error $RMSE^{u}$ of the subscriber’s *u* SKF estimated locations against the ground-truth (GT) locations obtained from GPS (Table 2).

**Figure 9:**
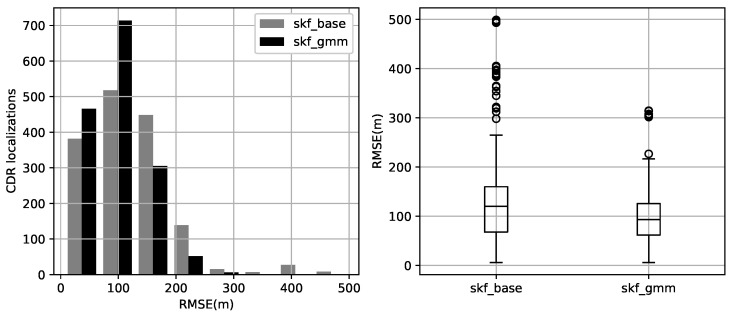
Evaluation of method’s localization accuracy using root means square error (RMSE) in meters between the ground-truth GPS location and method’s estimated location; baseline method *skf_base* using simple Gaussian to model the cell coverage vs. proposed method *skf_gmm* using Gaussian Mixtures.

The overall performance concerning our new model showed an increase in accuracy with a mean gain of 20% with a standard deviation reduction of 34%, as shown in Table 2. Moreover, the root means square error has decreased, and the error distribution has moved closer to zero as illustrated in Figure 9.

## 5. Discussion

**Figure 10:**
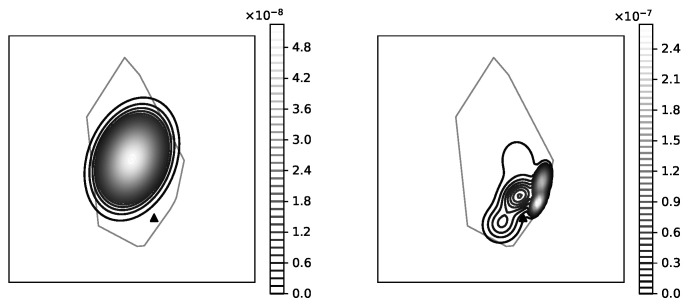
RF signal strength distribution approximated by *baseline* method (**left image**) and *proposed* one (**right image**). The *baseline* method estimates a multivariate normal distribution using the polygon shape only. The *proposed* method estimates a mixture of Gaussians using the samples of the RF signal strength within the polygon area.

The cellular coverage map (CM) is reflecting the best approximation of the area where a subscriber can expect an acceptable strength of the signal of a specific cellular transceiver. The CM is not a static map, but in fact best approximation of how the could look like at current time, taking into account the network load conditions. The best approximation is stored using raster image with fixed spatial resolution of pixels. Each pixel then represents the radio frequency (RF) signal strength at specific location (Figure 10). For the convenience further processing and visualization the RF raster data is converted into polygonal shapes and stored in vector shape files (SHP).

**Figure 11:**
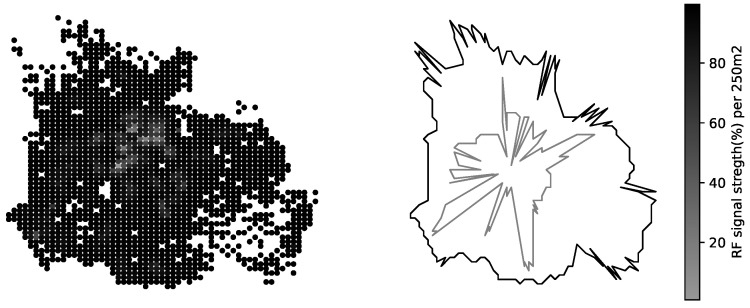
Cell coverage area represented by a raster map (**left one**) and a 2-level polygon (**right one**). Polygonal model provides only brief RF signal strength distribution within the cell coverage.

In vector format each RF transceiver is represented with exactly one polygon of probable coverage area. In addition vector format stores the important transceiver attributes like frequency, communication standard, the cell global identifier (CGI), mount elevation, radiation azimuth and sector width. Important aspect is that vector is the way the RF signal strength distribution is preserved in various formats of the coverage map. In raster format each pixel may have arbitrary value allowing to deliver a complex images of the RF signal distribution (Figure 10). In vector format RF signal strength values are averaged by a contour line of a polygon; inside the polygon the RF signal strength is at the level acceptable by a consumer equipment (reasonable quality of service); outside the polygon the RF signal strength is zero or very poor. Advanced vector formats use multiple polygons for a single transceiver; each polygon then represents a different RF signal strength level (Figure 11).

**Figure 12:**
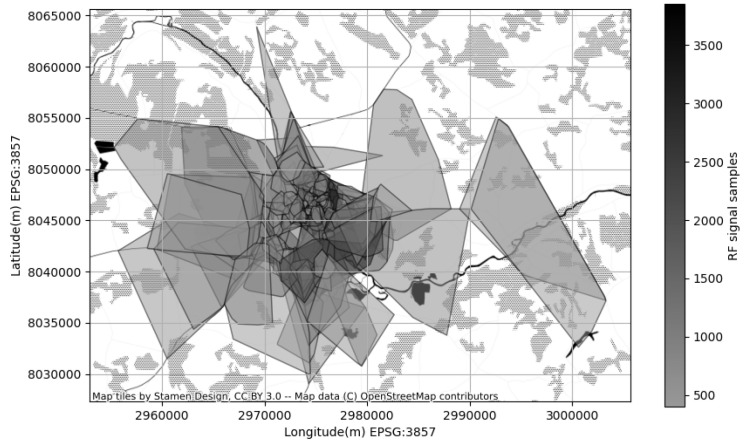
Coverage map modeled by 1-level polygons, allows to represent the overlaps of the cellular coverage areas; yet it does not allow to see the RF signal strength distribution within a cell’s polygon.

A clear advantage of vector format using polygons is the ability to show the overlaying nature of the RF signals of different transceivers; in cellular network a specific area (site) is normally covered by multiple RF signals of different frequency; the polygon representation illustrates its in much more comprehensible form (Figure 12).

The research suggest even more radical simplification of the coverage map representation; in particular the Voronoi diagram is derived from polygonal coverage map or from the raster map directly. Voronoi effectively cancels the RF overlay aspect of cellular network however it makes coverage map less realistic as it assumes sharp borderlines between the Voronoi cells of a coverage map; moreover, the shapes of the Voronoi cells are poorly matching those of original polygonal form or raster form. A compromise format that allows to model the RF signal strength distribution preserving RF overlay aspect and still be compact and simple is the use of multivariate Gaussian distribution N(μ,Σ), where $\mu \in R^{2}$ denotes the average location *longitude*, *latitude* and $\Sigma \in R^{2\times 2}$ is covariance matrix. Model’s naive assumption is the RF signal strength follows the normal distribution a therefore the signal is stronger in the middle of the coverage area and weaker on periphery. The corresponding values for μ and Σ can be obtained from polygonal model by taking polygon’s centroid as μ and by composing Σ using average radius of a polygon.

Alternatively, the parameters of the normal distribution can be estimated from the raster data directly using maximal likelihood estimation (MLE) method with a subsequent evaluation using Kolmogorov-Smirnov (KS) test. Modeling the RF signal coverage are using multivariate Gaussian variables allows to apply the state estimation and prediction method from control theory.

In particular, Kalman filter based method of estimating the subscriber location from CDR. The disadvantage of this kind of modeling the RF signal coverage is the shape limitation, namely the shape of the distribution is bound by an ellipse; however the actual shape might be more complex (according to raster coverage maps). To overcome the ellipse-shape limitation of the coverage modeling, an advanced modeling technique was suggested [38] using Gaussian Mixture Models (GMM) to grasp the complex shapes of coverage areas.

**Figure 13:**
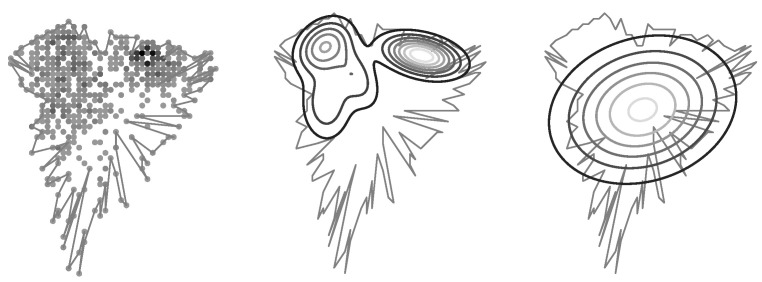
Initialization of Gaussian Mixture from cell’s raster map. The original cell’s polygon as well as the RF signal samples are shown on the left plot. The resulting mixture of Gaussian is shown on middle one. The right one illustrates the RF signal coverage distribution derived from the polygon only.

The initialization of GMM in this case is done directly from raster coverage maps. (Figure 13).

Initialization from polygonal coverage maps is also possible, but depends on how many signal strength levels are supported for a single transceiver represented by a polygon; the accuracy of a resulting GMM increases with them number of levels n>1. However, in case n=1 the polygonal model does not offer any information about the signal strength distribution; it only reflects where signal is at acceptable level. Therefore modeling the signal strength distribution using GMM does not offer any improvements contrast to Gaussian distribution in case of simple polygonal coverage maps.

The popularity of crowd collecting and the widespread of smartphones allowed to aggregate of accurate datasets of vector maps (OpenStreetMap) and cellular coverage RF signal samples (CellMapper). In scenarios where the CDR log is not featured with the coverage maps, the cell-specific RF signal patterns can be obtained through CellMapper, assuming the CDR log uses realistic CGIs (non-anonymous). Indeed the coverage maps obtained through CellMapper are not absolute. The RF signal samples are non-uniformly distributed and subject to the contributors’ mobility. It is impossible to get the RF signal samples in the areas where CellMapper’s contributors performed no measurements.

In scenarios where coverage map is not shared along with the CDR log, and the crowed collected repositories offer no samples, the RF attenuation pattern can be simulated based on cellular metadata. In their studies Hrovat et al. have focused on obtaining the raster signal coverage map based on the transceivers parameters [39]. The important ones included transceiver mount point exact location and elevation, antenna type, direction, sector width, signal frequency and transmitter power (dB); in addition an accurate digital elevation map (DEM) surrounding the transceiver mount is required. As a result of a simulation the raster coverage map is produced following spatial resolution of original DEM. A more general purpose open source RF analysis software “SPLAT!” (A Terrestrial RF Path Analysis Application For Linux/Unix: http://www.qsl.net/kd2bd/splat.html (accessed on 11 January 2023)) produces similar results.

In the end, using the Gaussian Mixture models in CDR-based localization increased the accuracy as demonstrated in Table 2. Compared to the baseline, the error has been reduced from miscalculations >500 m to <300 m. Moreover, those estimations with an error ranging between 100 and 200 m decreased to around 100 m error. Unfortunately, previous errors below 100m are not affected much by applying the new coverage modeling technique. The reason for such a limit of error reduction is fundamentally different ways of calculating the RF signal distribution from the coverage maps. The baseline method calculates the multivariate normal distribution based on a polygonal representation of the signal coverage; in particular, the μ and Σ are computed using the polygon’s centers and average radius under the assumption that the signal has normal distribution within the polygon. Accessing RF signal samples data associated with cell IDs revealed that large polygons have irregular signal distribution, often leaving most of the polygon with relatively poor RF signal level (Figure 10). In such conditions, Gaussian Mixture models describe the signal distribution much better, preserving the irregular patterns of the RF signal from the sampled raster maps. Smaller cells have more regular RF signal distribution and are well fit using multivariate normal of the baseline method; hence there is less difference in the errors compared to the proposed method.

## 6. Conclusions

The role of mobile devices in our daily life and how we interact with them made them perfect sensors for sensing urban mobility dynamics, population distribution, and displacement. With all the potential that mobile network data or CDR data offers still a couple of challenges to overcome due to the nature of data and its sparseness in time and space. Hence, this paper focuses on refining the quality of trajectories and mobility patterns extracted from CDR data by trying to perform mobile positioning. This increases the localization from a location area to a position like GPS.

Our approach explores using Gaussian Mixture models in CDR-based localization to increase accuracy. Our approach outcome and performance have demonstrated their ability to reduce the error in positioning mobile devices, which was reflected by a gain of 20% in accuracy compared to our baseline method. Our investigation clearly shows that it is essential to accurately estimate the coverage areas or RF to get a proper mobile positioning using CDR data.

Potential improvements in accuracy may be achieved by using the proposed method of applying GMMs to model the RF coverage and using the resulting distribution to source the PF particles. Studies of this kind would be a valuable contribution to continuing the idea of applying PF on CDR-based mobile positioning. In our current paper, we were not focusing on improvements in the positioning method (SKF), but rather on proofing that the proposed method of GMM coverage modeling contributes to accuracy improvement. Based on our results, it is clear that the GMM offers an advantage and should be investigated with other alternative approaches for future work. In addition, in the future, an alternative family of Conditional Markov switching hidden linear models (CMSHLM) should be considered as an alternative to SKF in MP based on CDR. The CMSHLMs can address most of the critical aspects the CDR-based MP is facing: (a) the non-linear motion, (b) non-gaussian error distribution, (c) the exact solution of KF filtering and smoothing.

## Figures and Tables

**Table 1:** Comparing the available datasets; important features are highlighted in separate columns: total number of unique subscribers; data collection period; recorded cell global identifiers (CGI); cellular coverage map; recorded GPS ground-truth data.

Dataset	Subscribers	Period	CGI	Coverage	GPS
MDC	200	1.5 years	anonymous	no	yes
CTU	1	142 days	yes	no	yes
D4D	9 mln.	1 year	no	no	no
RMD	100	125 days	yes	no	no
CDR’17	3	3 months	yes	yes	yes

**Table 2:** Root-mean-square error (meters) of localization methods *skf_base* original coverage estimation method compared to *skf_gmm* proposed method.

Method	Mean	Std	Max	25%	50%	75%
*skf_base*	123.54	73.47	499.08	68.0	120.23	160.0
*skf_gmm*	98.69	48.65	314.60	61.81	93.11	125.72
*Total gain:*	20%	34%	37%	9%	23%	21%
